# Supplementary material for: Anti-Trypanosoma cruzi Activity of Metabolism Modifier Compounds
Source: Int J Mol Sci. 2021 Jan 12;22(2):688. doi: 10.3390/ijms22020688 (PMC7828178; doi:10.3390/ijms22020688)
Supplement: Supplementary file 1 [file ijms-22-00688-s001.zip › ijms-1040263-supplementary/ijms-1040263-sup/Supplementary-Figure_3.docx]

**1** MPEETQTQDQPMEEEEVETFAFQAEIAQLMSLIINTFYSNKEIFLRELISNSSDALDKIR 60

**2** ---------------MTETFAFQAEINQLMSLIINTFYSNKEIFLRELISNSSDACDKIR 45

**3** ---------------MTETFAFQAEINQLMSLIINTFYSNKEIFLRELISNSSDACDKIR 45

**4** ---------------MTETFAFQAEINQLMSLIINTFYSNKEIFLRELISNSSDACDKIR 45

**5** ---------------MTETFAFQAEINQLMSLIINTFYSNKEIFLRELISNSSDACDKIR 45

**6** ---------------MTETFAFQAEINQLMSLIINTFYSNKEIFLRDVISNASDACDKIR 45

**7** ---------------MTETFAFQAEINQLMSLIINTFYSNKEIFLRELISNSSDACDKIR 45

.********* *******************::***:*** ****

**1** YESLTDPSKLDSGKELHINLIPNKQDRTLTIVDTGIGMTKADLINNLGTIAKSGTKAFME 120

**2** YQSLTNQAVLGDESHLRIRVVPDKANKTLTVEDTGIGMTKAELVNNLGTIARSGTKAFME 105

**3** YQSLTNQAVLGDESHLRVRVVPDKANKTLTVEDTGIGMTKAELVNNLGTIARSGTKAFME 105

**4** YQSLTNQAVLGDESHLRVRVIPDKANKTLTVEDTGIGMTKAELVNNLGTIARSGTKAFME 105

**5** YQSLTNQAVLGDESHLRIRVIPDKANKTLTVEDTGIGMTKAELVNNLGTIARSGTKAFME 105

**6** YQSLTDPSVLGDATRLCVRVVPDKENKTLTVEDNGIGMTKADLVNNLGTIARSGTKAFME 105

**7** YQSLTNQSVLGDEPHLRIRVIPDRVNKTLTVEDSGIGMTKADLVNNLGTIARSGTKSFME 105

*:***: : *.. .* :.::*:: ::***: *.*******:*:*******:****:***

**1** ALQAGADISMIGQFGVGFYSAYLVAEKVTVITKHNDDEQYAWESSAGGSFTVRTDTGEPM 180

**2** ALEAGGDMSMIGQFGVGFYSAYLVADRVTVVSKNNDDEAYTWESSAGGTFTVTPTPDCDL 165

**3** ALEAGGDMSMIGQFGVGFYSAYLVADRVTVVSKNNDDEAYTWESSAGGTFTVTPTPDCDL 165

**4** ALEAGGDMSMIGQFGVGFYSAYLVADRVTVVSKNNDDEAYTWESSAGGTFTVTPTPDCDL 165

**5** ALEAGGDMSMIGQFGVGFYSAYLVADRVTVVSKNNDDEAYTWESSAGGTFTVTPTPDCDL 165

**6** ALEAGADMSMIGQFGVGFYSAYLVADRVTVTSKNNSDEVYVWESSAGGTFTITSAPESDM 165

**7** ALEAGGDMSMIGQFGVGFYSAYLVADRVTVVSKNNEDDAYTWESSAGGTFTVTSTPDCDL 165

**:**.*:*****************::*** :*:*.*: *.*******:**: :

**1** GRGTKVILHLKEDQTEYLEERRIKEIVKKHSQFIGYPITLFVEKERDKEVSDDEAEEKED 240

**2** KRGTRIVLHLKEDQQEYLEERRLKDLIKKHSEFIGYDIELMVEKATEKEVTDEDEDEAAA 225

**3** KRGTRIVLHLKEDQQEYLEERRLKDLIKKHSEFIGYDIELMVEKATEKEVTDEDEDEAAA 225

**4** KRGTRIVLHLKEDQQEYLEERRLKDLIKKHSEFIGYDIELMVEKATEKEVTDEDEDEAAA 225

**5** KRGTRIVLHLKEDQQEYLEERRLKDLIKKHSEFIGYDIELMVEKATEKEVTDEDEDEAAA 225

**6** KLPARITLHLKEDQLEYLEARRLKELIKKHSEFIGYDIELMVEKTTEKEVTDEDEEEA-K 224

**7** KRGTRIVLHLKEDQQEYLEERRLKDLIKKHSEFIGYDIELMVENTTEKEVTDEDEDEEAA 225

::: ******* **** **:*:::****:**** * *:**: :***:*:: :*

**1** KEEEKEKEEKESEDKPEIEDVGSDEEEEKKDGDKKKKKKIKEKYIDQEELNKTKPIWTRN 300

**2** TKN-------EEGEEPKVEEVKDDAEE---GEKKKKTKKVKEVTQEFVVQNKHKPLWTRD 275

**3** TKN-------EEGEEPKVEEVKDDAEE---GEKKKKTKKVKEVTQEFVVQNKHKPLWTRD 275

**4** AKN-------EEGEEPKVEEVKDDAEE---GEKKKKTKKVKEVTQEFVVQNKHKPLWTRD 275

**5** AKN-------EEGEEPKVEEVKDDAEE---GEKKKKTKKVKEVTQEFVVQNKHKPLWTRD 275

**6** KAD-------EDGEEPKVEEVTEGEE-----GKKKKTKKVKEVTKEYEVQNKHKPLWTRD 272

**7** KKA-------EEGEEPKVEEVKDGVDA---DAKKKKTKKVKEVKQEFVVQNKHKPLWTRD 275

*. ::*::*:* .. : .***.**:** : ** **:***:

**1** PDDITNEEYGEFYKSLTNDWEDHLAVKHFSVEGQLEFRALLFVPRRAPFDLFENRKKKNN 360

**2** PKDVTKEEYAAFYKAISNDWEEPLSTKHFSVEGQLEFRAILFVPKRAPFDMFEPSKKRNN 335

**3** PKDVTKEEYAAFYKAISNDWEEPLSTKHFSVEGQLEFRAILFVPKRAPFDMFEPSKKRNN 335

**4** PKDVTKEEYAAFYKAISNDWEEPLSTKHFSVEGQLEFRAILFVPKRAPFDMFEPNKKRNN 335

**5** PKDVTKEEYAAFYKAISNDWEEPLSTKHFSVEGQLEFRAILFVPKRAPFDMFEPSKKRNN 335

**6** PKDVTKEEYAAFYKAISNDWEDPPATKHFSVEGQLEFRAIMFVPKRAPFDMLEPNKKRNN 332

**7** PKDVTKEEYASFYKAISNDWEEQLSTKHFSVEGQLEFRAILFLPKRAPFDMFEPNKKRNN 335

*.*:*:***. ***:::****: :.*************::*:*:*****::* **:**

**1** IKLYVRRVFIMDNCEELIPEYLNFIRGVVDSEDLPLNISREMLQQSKILKVIRKNLVKKC 420

**2** IKLYVRRVFIMDNCEDLCPEWLAFVRGVVDSEDLPLNISRENLQQNKILKVIRKNIVKKA 395

**3** IKLYVRRVFIMDNCEDLCPEWLAFVRGVVDSEDLPLNISRENLQQNKILKVIRKNIVKKA 395

**4** IKLYVRRVFIMDNCEDLCPEWLAFVRGVVDSEDLPLNISRENLQQNKILKVIRKNIVKKA 395

**5** IKLYVRRVFIMDNCEDLCPEWLAFVRGVVDSEDLPLNISRENLQQNKILKVIRKNIVKKA 395

**6** IKLYVRRVFIMDNCEDLCPDWLGFVKGVVDSEDLPLNISRENLQQNKILKVIRKNIVKKC 392

**7** IKLYVRRVFIMDNCEDLCPEWLGFLRGVVDSEDLPLNISRENLQQNKILKVIRKNIVKKA 395

***************:* *::* *::*************** ***.*********:***.

**1** LELFTELAEDKENYKKFYEQFSKNIKLGIHEDSQNRKKLSELLRYYTSASGDEMVSLKDY 480

**2** LELFEEIAENKEDYKKFYEQFGKNVKLGIHEDSANRKKLMELLRFHSSESGEDMTTLKDY 455

**3** LELFEEIAENKEDYKKFYEQFGKNVKLGIHEDSANRKKLMELLRFHSSESGEDMTTLKDY 455

**4** LELFEEIAENKEDYKKFYEQFGKNVKLGIHEDSANRKKLMELLRFHSSESGEDMTTLKDY 455

**5** LELFEEIAENKEDYKKFYEQFGKNVKLGIHEDSANRKKLMELLRFHSSESGEDMTTLKDY 455

**6** LEMFEEVAENKEDYKQFYEQFGKNIKLGIHEDTANRKKLMELLRFYSTESGEVMTTLKDY 452

**7** LELFEELAGNKEDYKKFYEQFSKNVKLGIHEDSTNRKKLMELLRFHSSESGEEMTTLKDY 455

**:* *:* :**:**:*****.**:*******: ***** ****:::: **: *.:****

**1** CTRMKENQKHIYYITGETKDQVANSAFVERLRKHGLEVIYMIEPIDEYCVQQLKEFEGKT 540

**2** VTRMKEGQKCIYYVTGDSKKKLETSPFIEQARRRGFEVLFMTEPIDEYVMQQVKDFEDKK 515

**3** VTRMKEGQKCIYYVTGDSKKKLETSPFIEQARRRGFEVLFMTEPIDEYVMQQVKDFEDKK 515

**4** VTRMKEGQKCIYYVTGDSKKKLETSPFIEQARRRGFEVLFMTEPIDEYVMQQVKDFEDKK 515

**5** VTRMKEGQKCIYYVTGDSKKKLETSPFIEQARRRGFEVLFMTEPIDEYVMQQVKDFEDKK 515

**6** VTRMKAEQNSIYYITGDSKKKLESSPFIEQAKRRGFEVLFMTEPYDEYVMQQVKDFEDKK 512

**7** VTRMKEGQKCIYYVTGDSKKKLETSPFIEQARRRGMEVLFMTDPIDEYVMQQVKEFEDKK 515

**** *: ***:**::*.:: .* *:*: :::*:**::* :* *** :**:*:**.*.

**1** LVSVTKEGLELPEDEEEKKKQEEKKTKFENLCKIMKDILEKKVEKVVVSNRLVTSPCCIV 600

**2** FACLTKEGVHFEETEEEKKQREEEKTAYERLCKAMKDVLSDKVEKVVVSERLATSPCILV 575

**3** FACLTKEGVHFEETEEEKKQREEEKTAYERLCKAMKDVLGDKVEKVVVSERLATSPCILV 575

**4** FACLTKEGVHFEETEEEKKQREEEKAAYERLCKAMKDVLGDKVEKVVVSERLATSPCILV 575

**5** FACLTKEGVHFEETEEEKKQREEEKTAYERLCKAMKDVLGDKVEKVVVSERLATSPCILV 575

**6** FACLTKEGVHFEESEEEKRQREEEKATCEKLCKTMKEVLGDKVEKVTVSERLSTSPCILV 572

**7** FACLTKEGVHFEETEEEKKQREEEKASYERLCKAMKEVLGDKVEKVVVSDRLATSPCILV 575

:..:****:.: * ****:::**:*: *.*** **::* .*****.**:** **** :*

**1** TSTYGWTANMERIMKAQALRDNSTMGYMAAKKHLEINPDHSIIETLRQKAEADKNDKSVK 660

**2** TSEFGWSAHMEQIMRNQALRDSSMSAYMMSKKTMEINPAHPIVKELKRRVEADENDKAVK 635

**3** TSEFGWSAHMEQIMRNQALRDSSMSAYMMSKKTMEINPAHPIVKELKRRVEADENDKAVK 635

**4** TSEFGWSAHMEQIMRNQALRDSSMSAYMMSKKTMEINPAHPIVKELKRRVEADENDKAVK 635

**5** TSEFGWSAHMEQIMRNQALRDSSMSAYMMSKKTMEINPAHPIVKELKRRVEADENDKAVK 635

**6** TSEFGWSAHMEQMMRNQALRDSSMAQYMMSKKTMELNPKHPIIKELRRRVEADENDKAVK 632

**7** TSEFGWSAHMEQIMRNQALRDSSMSAYMMRKKTMEINTTHAIVKELKRRVEADENDKAAK 635

** :**:*:**::*: *****.* ** ** :*:* * *:: *:::.***:***:.*

**1** DLVILLYETALLSSGFSLEDPQTHANRIYRMIKLGLGIDEDDPTADDTSAAVTEEMPPLE 720

**2** DLVYLLFDTALLTSGFTLDDPTSYAERIHRMIKLGLSLDDEDNGNEEAEP--A-AAVPAE 692

**3** DLVYLLFDTALLTSGFTLDDPTSYAERIHRMIKLGLSLDDEDNGNEEAEP--A-AAVPAE 692

**4** DLVYLLFDTALLTSGFTLDDPTSYAERIHRMIKLGLSLDDEDNGNEESEP--A-AAVPAE 692

**5** DLVYLLFDTALLTSGFTLDDPTSYAERIHRMIKLGLSLDDEDNGNEEAEP--A-AAVPAE 692

**6** DLVFLLFDTSLLTSGFQLEDPT-YAERINRMIKLGLSLDEEEEEEAVEAA--VAETAPAE 689

**7** DLIFLLFDTSLLTSGFTLDDPTAYADRIHRMIKLGLSLDDDAEEEEAQAP--VAAA-AAN 692

**: **::*:**:*** *:** :*:** *******.:*:: . : :

**1** GDDDTSRMEEVD 732

**2** PVAGTSSMEQVD 704

**3** PVAGTSSMEQVD 704

**4** SVAGTSSMEQVD 704

**5** PVAGTSSMEQVD 704

**6** VTAGTSSMELVD 701

**7** SSTGASGMEEVD 704

.:* ** **

**Supplementary Figure 3.** CLUSTAL O (version 1.2.4) multiple sequence alignment of human and Kinetoplastid parasites Hsp90 protein sequences. Bold numbers on the left indicate: **1-** Human Hsp90α isoform 2 (NCBI ref.: NP_005339.3); **2-** *T. cruzi* Dm28c Hsp83 (TriTrypDB ref.: C4B63_113g33); **3-** *T. cruzi* Sylvio X10/1 Hsp 83 (TriTrypDB ref.: TcSYL_0008070); **4-** *T. cruzi* marinkellei strain B7 Hsp83 (TriTrypDB ref.: Tc_MARK_3581); **5-** *T. cruzi* TCC Hsp83 (TriTrypDB ref.: C3747_167g6); **6-** *L. amazonensis* Hsp83 (NCBI ref.: AAA29250.1); **7-** *T. brucei* Hsp83 (NCBI ref.: XP_823307.1). In reference sequence **1**: N-terminal domain is highlighted in grey; middle domain in blue; C-terminal domain in green and the charge domain in purple. Those residues reported to interact with 17-DMAG are shown in yellow (direct and water-mediated interactions). Numbers on the right side of each line indicate the residue position within each sequence. These were obtained from the NCBI and TriTrypDB public databases [57,58].
