# Supplementary material for: Anti-Trypanosoma cruzi Activity of Metabolism Modifier Compounds
Source: Int J Mol Sci. 2021 Jan 12;22(2):688. doi: 10.3390/ijms22020688 (PMC7828178; doi:10.3390/ijms22020688)
Supplement: Supplementary file 1 [file ijms-22-00688-s001.zip › ijms-1040263-supplementary/ijms-1040263-sup/Supplementary_Figure_2.docx]

**
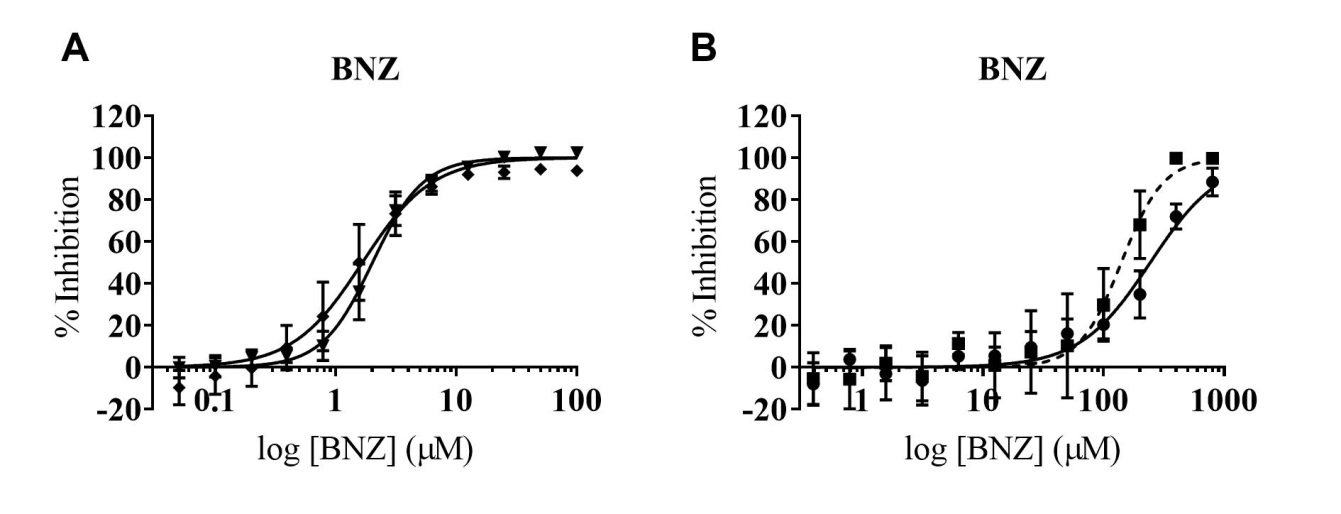
**

**Supplementary Figure S2**. BNZ Dose-Response Curves. **A**) Anti-*T. cruzi* assays: anti-*T. cruzi* activity of BNZ in the “screening” assay is represented by rhombus, and its anti-amastigote specific activity by triangles. **B**) BNZ toxicity on Vero cells: AlamarBlue-based readout data is represented by circles and a straight line whereas crystal violet-based readout data is represented by squares and a dashed line.
