# Supplementary material for: Anti-Trypanosoma cruzi Activity of Metabolism Modifier Compounds
Source: Int J Mol Sci. 2021 Jan 12;22(2):688. doi: 10.3390/ijms22020688 (PMC7828178; doi:10.3390/ijms22020688)
Supplement: Supplementary file 1 [file ijms-22-00688-s001.zip › ijms-1040263-supplementary/ijms-1040263-sup/Supplementary_Table_1.docx]

**Scheme 1.** Average IC_50_ Values of Non-Progressed Compounds.

| Compound | IC50 (µM) |
| --- | --- |
| Oligomycin | 0.52 |
| Etomoxir | 41.22 |
| SC79 | 115.8 |
| Akti-1/2 | N.A. |
| AICAR | 140.4 |
| Doxycycline | 50.85 |
| Sodium salicylate | N.A. |

N.A., not adjusted.
