# Supplementary figures and images for: Anti-Trypanosoma cruzi Activity of Metabolism Modifier Compounds
Source: Int J Mol Sci. 2021 Jan 12;22(2):688. doi: 10.3390/ijms22020688 (PMC7828178; doi:10.3390/ijms22020688)

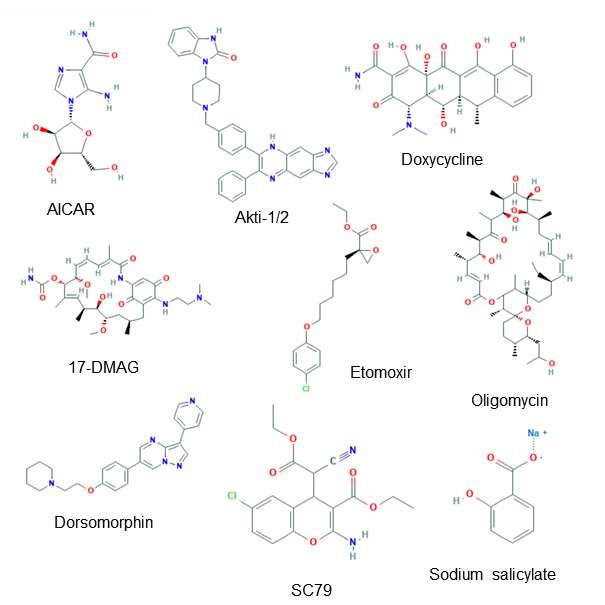


**Supplementary Figure S1**. Chemical Structures of the Compounds/Drugs Evaluated in this Work.

Supplement: Supplementary file 1 [file ijms-22-00688-s001.zip › ijms-1040263-supplementary/ijms-1040263-sup/Supplementary_Figure_1.docx]
